# Nuclear factor‐kappa B‐dependent X‐box binding protein 1 signalling promotes the proliferation of nucleus pulposus cells under tumour necrosis factor alpha stimulation

**DOI:** 10.1111/cpr.12542

**Published:** 2018-11-14

**Authors:** Lu Chen, Zhi‐Yang Xie, Lei Liu, Lei Zhu, Feng Wang, Pan Fan, Arjun Sinkemani, Cong Zhang, Xin Hong, Xiao‐Tao Wu

**Affiliations:** ^1^ Department of Spine Surgery, Zhongda Hospital, School of Medicine Southeast University Nanjing China

**Keywords:** NF‐κB, nucleus pulposus, proliferation, TNF‐α, unfolded protein response

## Abstract

**Objectives:**

Tumour necrosis factor alpha (TNF‐α) expressed by nucleus pulposus cells (NPCs) plays a critical role in intervertebral disc (IVD) degeneration. A key unfolded protein response (UPR) component, X‐box binding protein 1 (XBP1) and nuclear factor‐kappa B (NF‐κB) are essential for cell survival and proliferation. The aim of our study was to elucidate the roles of XBP1 and NF‐κB in IVD degeneration (IDD).

**Materials and methods:**

Rat NPCs were cultured with TNF‐α in the presence or absence of XBP1 and NF‐κB‐p65 small interfering RNA. The associated genes and proteins were evaluated through quantitative real‐time PCR, Western blot analyses and immunofluorescence staining to monitor UPR and NF‐κB signalling and identify the regulatory mechanism of p65 by XBP1. Cell counting kit‐8 assay, cell cycle analysis and related gene and protein expression were performed to examine the proliferation of NPCs.

**Results:**

The acute exposure of TNF‐α accelerated the proliferation of rat NPCs by activating the UPR/XBP1 pathway. XBP1 signalling favoured the phosphorylation and nuclear translocation of p65 subunit of NF‐κB. The activation of NF‐κB in the later phase also enhanced NPC proliferation.

**Conclusions:**

Unfolded protein response reinforces the survival and proliferation of NPCs under TNF‐α stimulation by activating the XBP1 pathway, and NF‐κB serves as a vital mediator in these events. The XBP1 signalling of UPR can be a novel therapeutic target in IDD.

## 1 INTRODUCTION

Low back pain (LBP) caused by intervertebral disc (IVD) degeneration has been a common clinical disease in the orthopaedic department. LBP has become a serious health and socioeconomic problem affecting modern society.^1^ Among numerous degeneration generators in the lumbar spine, the cellular loss of nucleus pulposus cells (NPCs) is considered the most important contributor to IVD degeneration (IDD).^2^

The pro‐inflammatory cytokine TNF‐α has been strongly linked to disc pathology and implicated in painful IDD. TNF‐α expression increases with age and IDD severity.^3^ In the onset of IDD, TNF‐α initiates NPC apoptosis and extracellular matrix (ECM) degradation. However, our previous studies demonstrated that TNF‐α enhances the proliferation of human NPCs through nuclear factor‐kappa B (NF‐κB), c‐Jun N‐terminal kinase (JNK) and p38 mitogen‐activated protein kinase (p38 MAPK) pathways.^4^ In view of this contradictory phenomenon, we supposed that different cell stress statuses lead to different biological changes of apoptosis or proliferation NPCs under TNF‐α stimulation. And whether a healthy IVD can recover after its exposure to TNF‐α remains uncertain.^5^ These matters all need to be further clarified in our study.

Nuclear factor‐kappa B is vital for genes involved in immune response, inflammation, cell survival, adhesion and proliferation.^6^ The canonical NF‐κB signalling has been considered the central mediator of the inflammatory process.^7^ Inflammation and NF‐κB have a double‐edged role in cells. On the one hand, activation of NF‐κB is part of the immune defence, which usually results in the upregulation of anti‐apoptotic genes thereby providing cell survival mechanism to withstand the physiological stress that triggered the inflammatory response.^8^ Under normal conditions, NF‐κB plays a critical role in protecting cells against apoptosis,^9^ stimulating cell proliferation^10^ and promoting cell migratory. On the other hand, persistent and aberrant activation of NF‐κB stimulates IVD cells to synthesize many pro‐inflammatory cytokines (such as TNF‐α, IL‐1, IL‐6 and IL‐8) to amplify inflammatory responses. Moreover, NF‐κB signalling was shown to contribute to the upregulation of matrix metalloproteinases (MMPs) and a disintegrins and metalloproteinases with thrombospondin motifs (ADAMTSs), which exacerbate the loss of ECM, especially aggrecan (ACAN) and collagen Ⅱ (COL2), thereby aggravating the degeneration of IVD.^8^ Clearly, either too little or too much NF‐κB activity is detrimental; furthermore, NF‐κB signalling pathway is also crucial in maintaining a healthy homeostasis.

Nuclear factor‐kappa B homodimers and heterodimers are formed by five family members, namely p65 (RelA), p50, p52, RelB and c‐Rel, and the most canonical form is the p65/p50 heterodimer.^11^ The activity of these dimers is regulated by an inhibitory protein known as the inhibitor of κB (IκB).^12^, ^13^ As a pro‐inflammatory cytokine, TNF‐α can activate NF‐κB signalling. In resting cells, the inactive form of NF‐κB is sequestered in the cytoplasm combined with IκB. Extracellular stimuli, such as TNF‐α, activate the phosphorylation and proteolytic degradation of IκB. The released p65/p50 then rapidly translocates to the nucleus and regulates the transcription of target genes, including cytokines, cell adhesion molecules, hematopoietic growth factors and prosurvival genes.^14^

Endoplasmic reticulum (ER) performs important cell functions and plays a crucial role in the folding of newly synthesized proteins.^15^ ER stress elicits an unfolded protein response (UPR) to facilitate repair and thus reestablish normal ER functioning through the mediation of three ER‐resident transducers, namely inositol‐requiring enzyme 1 (IRE1), protein kinase RNA‐like ER kinase (PERK) and activating transcription factor 6 (ATF6).^16^

Various agents inducing ER stress and UPR activation can trigger NF‐κB signalling.^17^ The activated NF‐κB can translocate to the nucleus and stimulate target gene transcription. As such, a direct ER‐nuclear signal transduction pathway is established.^18^ Although studies have yet to fully explain the underlying mechanism of NF‐κB activation in UPR, studies have revealed that the activation of NF‐κB can be induced by PERK‐mediated eIF2α phosphorylation, XBP1 splicing, IRE1 phosphorylation, oxidative stress and calcium disturbances.^13^, ^19^

Many scholars found that TNF‐α has extensive connections with ER stress and UPR.^20^, ^21^, ^22^ In addition, our previous studies demonstrated that TNF‐α activates ER stress, and the consequent initiation of UPR reinforces the survival and proliferation of NPCs.^23^ Considering the importance of NF‐κB in immunity, inflammation, anti‐apoptosis and cell proliferation,^12^ we hypothesized that UPR exerts cytoprotection and promotes NPC proliferation under TNF‐α stimulation by activating NF‐κB signalling. In the current research, we conducted experiments to verify whether UPR could trigger the NF‐κB signalling pathway in NPCs, the function of NF‐κB signalling and its association with UPR in TNF‐α‐induced biological changes in NPCs. Our data suggested that the NF‐κB‐dependent XBP1 signalling pathway represented an adaptive mechanism that prevented NPC apoptosis and promoted cell proliferation under TNF‐α stimulus.

## 2 MATERIALS AND METHODS

### 2.1 Cell isolation and culture

Nucleus pulposus cells were collected from the tail discs of Sprague Dawley rats in accordance with previously described methods,^24^ resuspended in DMEM/F12 containing 10% FBS and antibiotics and incubated at 37°C with 5% CO_2_ in a humidified incubator. The cells after the third passage were identified based on the newly defined NPC phenotype^25^ and used in the subsequent studies (Figure S1). The experimental protocol was approved by the Institutional Animal Care and Use Committee of Southeast University (Nanjing, China).

### 2.2 Small interfering RNA transfection

Transfection was performed in accordance with the manufacturer's protocol. In brief, the cells cultured in six‐well round‐bottomed plates were transfected with XBP1 small interfering RNA (siRNA; GenePharma, Shanghai, China) or p65 siRNA (GenePharma) by using Lipofectamine RNAiMAX reagent in Opti‐MEM medium (Invitrogen, Carlsbad, CA, USA). The sequences of the specific siRNAs were as follows: XBP1, sense (5′‐3′) CUGCUAAUCUGGAGGAACUTT; antisense (5′‐3′) AGUUCCUCCAGAUUAGCAGTT; NF‐κB‐p65, sense (5′‐3′) GCUUUGACUCACUCCAUAUTT; and antisense (5′‐3′) AUAUGGAGUGAGUCAAAGCTT. A nontargeting siRNA was used as a control. The medium was replaced with a fresh one, and the NPCs were harvested for the subsequent experiments after 24 or 48 hours.

### 2.3 Cell proliferation assay

Cell proliferation was quantified using a cell counting kit‐8 (CCK‐8; KeyGen, Nanjing, China). The NPCs were cultured in 96‐well plates at a density of 2000 cells/well with or without siRNA transfection. Then, each well was added with CCK‐8 reagent at various time points and incubated at 37°C in 5% CO_2_ in air atmosphere for 4 hours. Absorbance at 450 nm was detected with Multiskan MK3 (Thermo Scientific, Waltham, MA, USA) to calculate cell viability.

### 2.4 Immunofluorescence staining

Nucleus pulposus cells were seeded into 24‐well plates at 8000 cells per well and allowed to adhere to the glass bottom for 24 hours. The cells with or without siRNA interference were treated using TNF‐α (10 ng/mL), fixed with 4% paraformaldehyde at room temperature and permeabilized with 0.3% Triton X‐100. The treated cells were incubated with rabbit Ki‐67 (Abcam, ab15580, Cambridge, MA, USA; 1:500), rabbit phosphor‐p65 (*p*‐p65, Abcam, ab86299; 1:500) or mouse XBP1 (Invitrogen, MA5‐15768; 1:500) at 4°C overnight. Consequently, the cells were stained with the respective secondary antibodies Alexa Fluor 647 goat anti‐rabbit IgG (Abcam, ab150079; 1:1000) and Alexa Fluor 488 goat anti‐mouse IgG (Abcam, ab150113; 1:1000). Nuclei were counterstained with DAPI and observed under a fluorescence microscope (Olympus, Tokyo, Japan).

### 2.5 Cell cycle analysis through flow cytometry

The NPCs were harvested through trypsinization, fixed with 70% cold ethanol for more than 2 hours, centrifuged, resuspended in 50 μL of RNase A, incubated for 30 minutes at 37°C and added with 450 μL of propidium iodide (KeyGen) in the dark. The samples were then analysed using a BD FACSCanto Ⅱ flow cytometer (BD Biosciences, San Jose, CA, USA).

### 2.6 Quantitative real‐time PCR (qRT‐PCR) analysis

Total cellular RNA was isolated with TRIzol reagent (Invitrogen). Purified RNA was reverse transcribed using PrimeScript^TM^ RT Master Mix (TaKaRa, RR036A, Dalian, China). The primer sequences are presented in Table 1. Gene transcripts were quantified using SYBR Premix EX Taq^TM^ (TaKaRa, RR420A) through real‐time PCR (Applied Biosystems, Foster City, CA, USA), and relative gene expression was measured using the 2^−ΔΔ^ *^Ct^* method with β‐actin gene as an internal control.

**Table 1:** Primers sequences for quantitative real‐time PCR

Gene	Forward (5′‐3′)	Reverse (5′‐3′)
Cyclin D1	TCAAGTGTGACCCGGACTG	GACCAGCTTCTTCCTCCACTT
Cyclin B1	TGAACTTCAGTCTGGGTCGC	GGCAAAATGCACCATGTCGT
XBP1	AGTTAAGGACACGCTTGGGG	CACGTAGTCTGAGTGCTGCG
p65	CATGGATCCCTGCACACCTT	CTCAGCATGGAGAGTTGGCA
β‐actin	CACCCGCGAGTACAACCTTC	CCCATACGCACCATCACACC

qRT‐PCR, quantitative real‐time polymerase chain reaction.

### 2.7 Western blot analysis

Total protein extracts from NPCs were obtained through a whole‐cell lysis assay (KeyGen). Protein concentration was determined with a BCA assay (Beyotime, Shanghai, China). Proteins were separated with 10% SDS‐PAGE and transferred onto PVDF membranes. The membranes were blocked with 5% skimmed milk in TBST for 1.5 hours and incubated with primary antibodies at 4°C overnight (Table 2). The membranes were then incubated with secondary antibodies (Abcam, ab6721; 1:5000) diluted in 5% bovine serum albumin (BSA)/TBST at room temperature for 2 hours. Bands were detected and assessed through densitometric analysis. Protein expression level was normalized to that of vinculin.

**Table 2:** Primary antibodies for Western blot

Target	Antibody (company, catalog number, usage)
BiP	Abcam, ab21685 (1:1000)
*p*‐eIF2α	Abcam, ab32157 (1:500)
*t*‐eIF2α	Abcam, ab131495 (1:500)
XBP1	Abcam, ab37152 (1:500)
Cyclin D1	Abcam, ab134175 (1:10000)
Cyclin B1	Proteintech (Chicago, IL, USA), 55004‐1‐AP (1:1000)
*p*‐p65	Abcam, ab86299 (1:1000)
p65	Abcam, ab16502 (1:1000)
Aggrecan	Abcam, ab36861 (1:1000)
Collagen Ⅱ	Abcam, ab34712 (1:1000)
Vinculin	Abcam, ab129002 (1:10000)

### 2.8 Statistical analysis

Data were presented as mean ± SD of at least three independent experiments. Statistical analyses were carried out using GraphPad Prism 6 (GraphPad Software, Inc, La Jolla, CA, USA). Two‐tailed Student's *t* test was conducted to compare differences among two groups, and one‐way ANOVA was conducted to compare differences among multiple groups. *P*‐values <0.05 were considered statistically significant.

## 3 RESULTS

### 3.1 TNF‐α promoted the proliferation of NPCs

**Figure 1:**
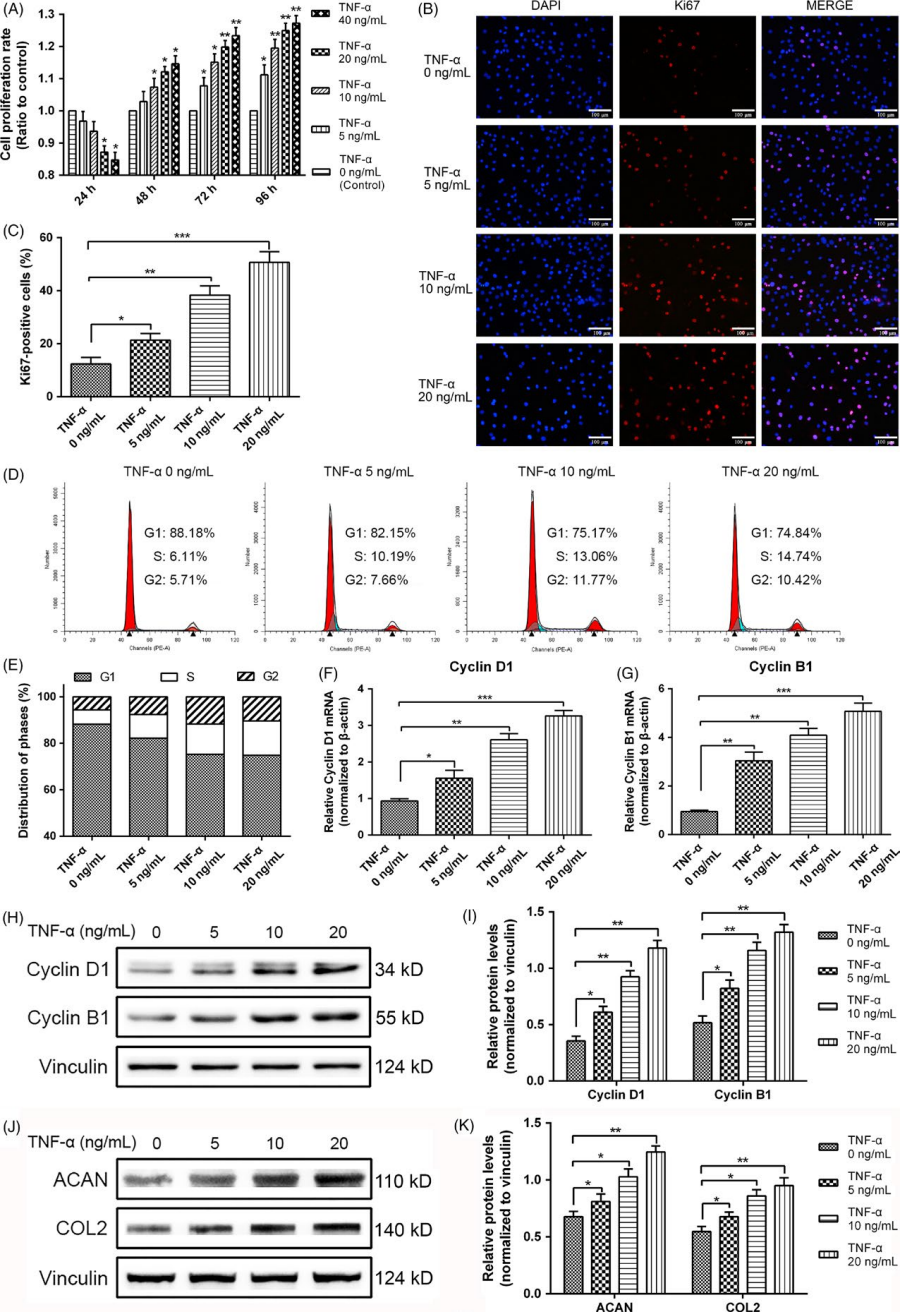
Tumour necrosis factor alpha (TNF‐α) enhanced the proliferation of rat nucleus pulposus cells (NPCs). A, Cell proliferation was evaluated through CCK‐8 analysis at different time points upon exposure to gradient concentrations of TNF‐α (5, 10, 20 and 40 ng/mL). B, NPC proliferation under TNF‐α (0, 5, 10 and 20 ng/mL) stimulation for 24 h was measured through Ki67 immunofluorescence staining (original magnification, ×200). C, Ki67‐positive cells were quantified upon exposure to different TNF‐α concentrations at 24 h. D,E, The proportions of cells in each cycle were measured through flow cytometry with different concentrations of TNF‐α stimulation at 24 h. F,G, After TNF‐α treatment (0, 5, 10 and 20 ng/mL) for 24 h, the mRNA expression levels of cyclin D1 and cyclin B1 were detected through qRT‐PCR and normalized to that of β‐actin. H,I, Cyclin D1 and cyclin B1 expression levels in NPCs subjected to different treatments were measured through Western blot analysis and normalized to that of vinculin. J,K, ACAN and COL2 expression levels in NPCs subjected to different treatments were measured through Western blot analysis and normalized to that of vinculin. The results were presented as mean ± SD (*n* = 3). *P < 0.05; **P < 0.01; ***P < 0.001

The rat NPCs were treated with a gradient TNF‐α concentration to explore the biological effects of TNF‐α on the proliferation of NPCs. CCK‐8 assay was performed to calculate the rate of cell proliferation. After 24 hours, the proliferation rate of NPCs increased upon exposure to the TNF‐α stimulus (Figure 1A). To further validate the role of TNF‐α on the proliferation of rat NPCs, we subjected the proliferating relevant antigen Ki67 to immunofluorescence staining and conducted cell cycle analysis. The percentage of Ki67‐positive NPCs increased significantly under TNF‐α stimulation compared with that of the control group (Figure 1B,C). Moreover, TNF‐α promoted more cells to transit to the S phase and decreased the percentage of the cells arrested in the G1 phase (Figure 1D,E), indicating that TNF‐α was positively correlated with NPC proliferation. The gene and protein levels of cyclin D1 and cyclin B1 were measured through qPCR and Western blot analyses, respectively. The expression of cyclin D1 and cyclin B1 notably increased at mRNA and protein levels by the gradient TNF‐α concentration after 24 hours. This finding indicated the rapid proliferation of the surviving cells (Figure 1F‐I). To further investigate the function of proliferous NPCs, we measured the protein levels of ACAN and COL2 through Western blot analyses (Figure 1J,K). The expression of ACAN and COL2 increased accompanied by the proliferation of NPCs under TNF‐α stimulus. This finding indicated that proliferous NPCs were capable in ECM synthesis, which in turn confirmed the proliferative viability of the cells. Hence, acute TNF‐α stimulation induced the proliferation of rat NPCs.

### 3.2 TNF‐α activated UPR signalling and XBP1 signalling could be silenced by XBP1 siRNA

**Figure 2:**
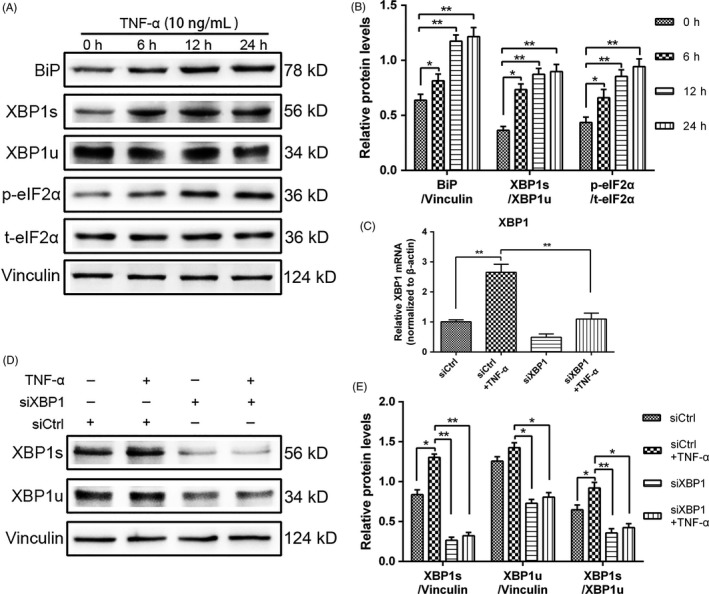
Activation of unfolded protein response (UPR) signalling after tumour necrosis factor alpha (TNF‐α) treatment in rat nucleus pulposus cells (NPCs) and X‐box binding protein 1 (XBP1) siRNA inhibition of the TNF‐α‐induced activation of the XBP1 pathway. A, After treatment with 10 ng/mL TNF‐α, the activation of UPR signalling was assessed in terms of the related protein expression at different time intervals (0, 6, 12 and 24 h). B, UPR markers in NPCs were analysed through Western blot, and the protein expression levels of BiP were normalized to that of vinculin. The protein expression levels of *p*‐eIF2α and XBP1s were normalized to those of total eIF2α (*t*‐eIF2α) and XBP1u. NPCs were treated with control siRNA (siCtrl) or XBP1 siRNA (siXBP1) for 24 h and subsequently exposed to 10 ng/mL TNF‐α for another 24 h. C, The mRNA expression of XBP1 in NPCs was quantified through qRT‐PCR. The mRNA expression levels were normalized to that of β‐actin. D,E, XBP1s was evaluated through Western blot, and the protein expression levels were normalized to those of XBP1u. The results were presented as mean ± SD (*n* = 3). *P < 0.05; **P < 0.01

Our previous studies showed that the proportion of apoptotic cells increases at the early stage when TNF‐α concentrations exceed 20 ng/mL.^23^ Therefore, we selected a TNF‐α concentration of 10 ng/mL for the subsequent experiments. The NPCs were treated with 10 ng/mL TNF‐α at different time points. Using Western blot, we examined the binding immunoglobulin protein (BiP) expression, eIF2α phosphorylation by using an antibody specific to the phosphorylated form of eIF2α (*p*‐eIF2α) and the spliced form of XBP1 (XBP1s), which is an important regulator of the UPR. The protein levels of UPR markers increased after 6 hours and were considerably elevated by TNF‐α stimulus at 12 and 24 hours (Figure 2A,B). These results indicated that TNF‐α activated the UPR signalling pathway. To confirm that UPR/XBP1 pathway could be silenced in these cells through siRNA transfection, we use XBP1 siRNA to knock down XBP1 signalling. The inhibition efficiency was verified by the decreased gene and protein expression of XBP1 compared with that of the negative control. The mRNA expression of XBP1 in the siRNA transfection group decreased compared with that of the negative siRNA group (Figure 2C). Consistent with the result of qRT‐PCR, our findings indicated that the TNF‐α‐induced increase in the protein expression of XBP1s and XBP1u could be silenced by XBP1 siRNA. The value of XBP1s/XBP1u decreased with XBP1 siRNA interference (Figure 2D,E).

### 3.3 UPR signalling regulated the TNF‐α‐induced proliferation of NPCs through XBP1 signalling

**Figure 3:**
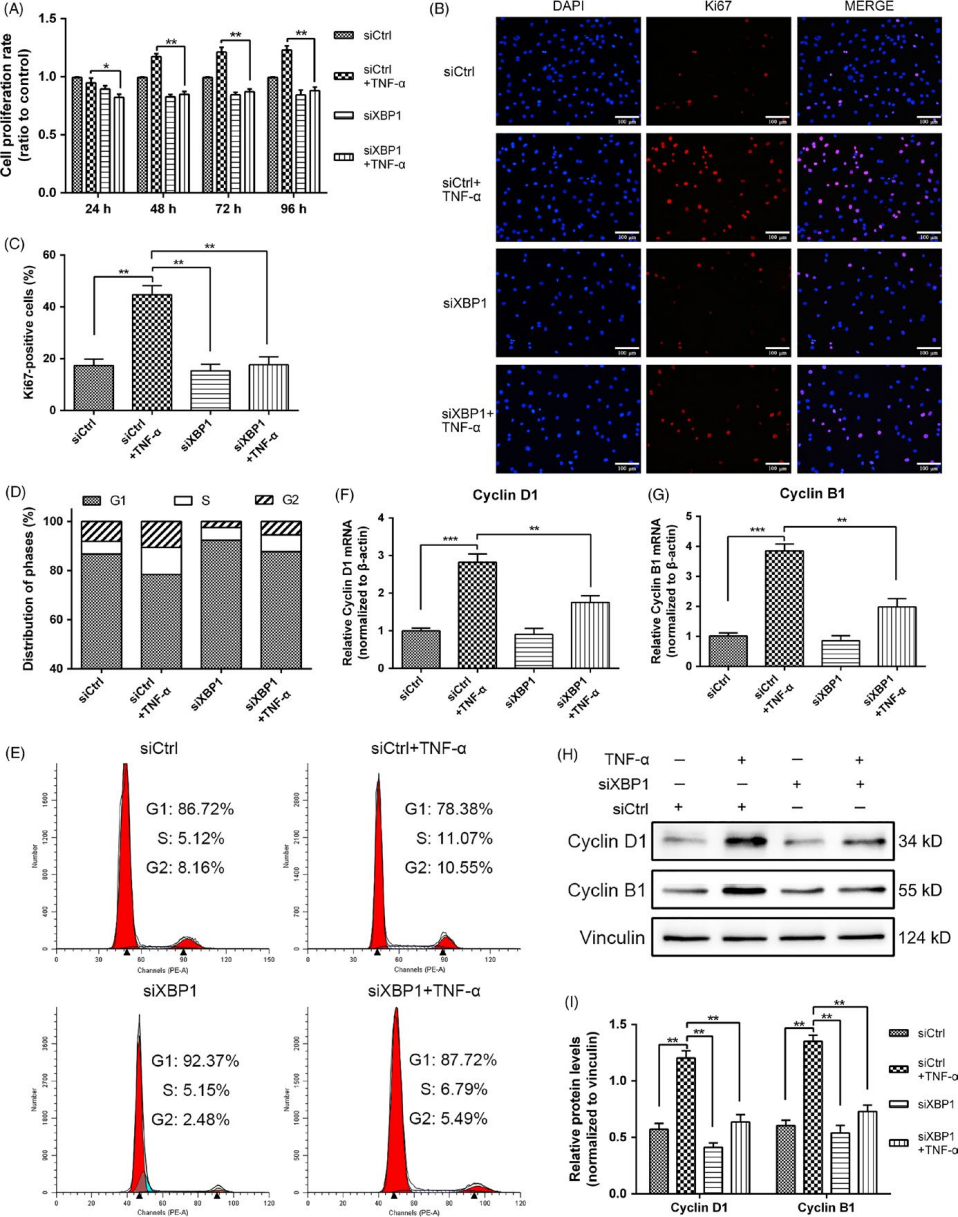
Role of inositol‐requiring enzyme 1/X‐box binding protein 1 (XBP1) pathway in rat NPC proliferation induced by tumour necrosis factor alpha (TNF‐α). Nucleus pulposus cells (NPCs) were treated with siCtrl or siXBP1. At 24 h post‐transfection, the cells were treated with 10 ng/mL TNF‐α for additional 24 h. A, Cell proliferation was evaluated through CCK‐8 analysis at different time points. B,C, The proliferating antigen Ki67 was subjected to immunofluorescence staining (original magnification, ×200), and Ki67‐positive cells were quantified. D,E Cell cycle distribution was measured through flow cytometry. F,G, The mRNA expression levels of cyclin D1 and cyclin B1 were detected through qRT‐PCR and normalized to that of β‐actin. H,I, Cyclin D1 and cyclin B1 expression levels were measured through Western blot analysis in NPCs subjected to different treatments and normalized to the expression level of vinculin. The results were presented as mean ± SD (*n* = 3). *P < 0.05; **P < 0.01; ***P < 0.001

To investigate the effect of UPR signalling on NPC proliferation induced by TNF‐α, we silenced the XBP1 signalling of UPR by using siRNA. After the treatment with TNF‐α (10 ng/mL) or siRNA specific to XBP1 was administered, the proliferation rate of NPCs was measured through CCK‐8 assay, and XBP1 silencing decreased the TNF‐α‐induced NPC proliferation at different time points (Figure 3A). The treatment with XBP1 siRNA decreased the percentage of Ki67‐positive NP cells (Figure 3B,C), indicating a positive effect of XBP1 signalling on NPC proliferation. As expected, the cell cycle analysis showed that the proportion of the cells in the S phase in the XBP1 interference group decreased, and the percentage of the cells arrested in the G1 phase increased (Figure 3D,E). The gene and protein expression levels of cyclin D1 and cyclin B1 were detected through qPCR (Figure 3F,G) and Western blot analyses (Figure 3H,I). The results revealed that the knockdown of XBP1 significantly decreased the cyclin D1 and cyclin B1 expression. Hence, UPR signalling regulated the TNF‐α‐induced proliferation of NPCs through XBP1 signalling.

### 3.4 UPR initiated the secondary activation of NF‐κB

**Figure 4:**
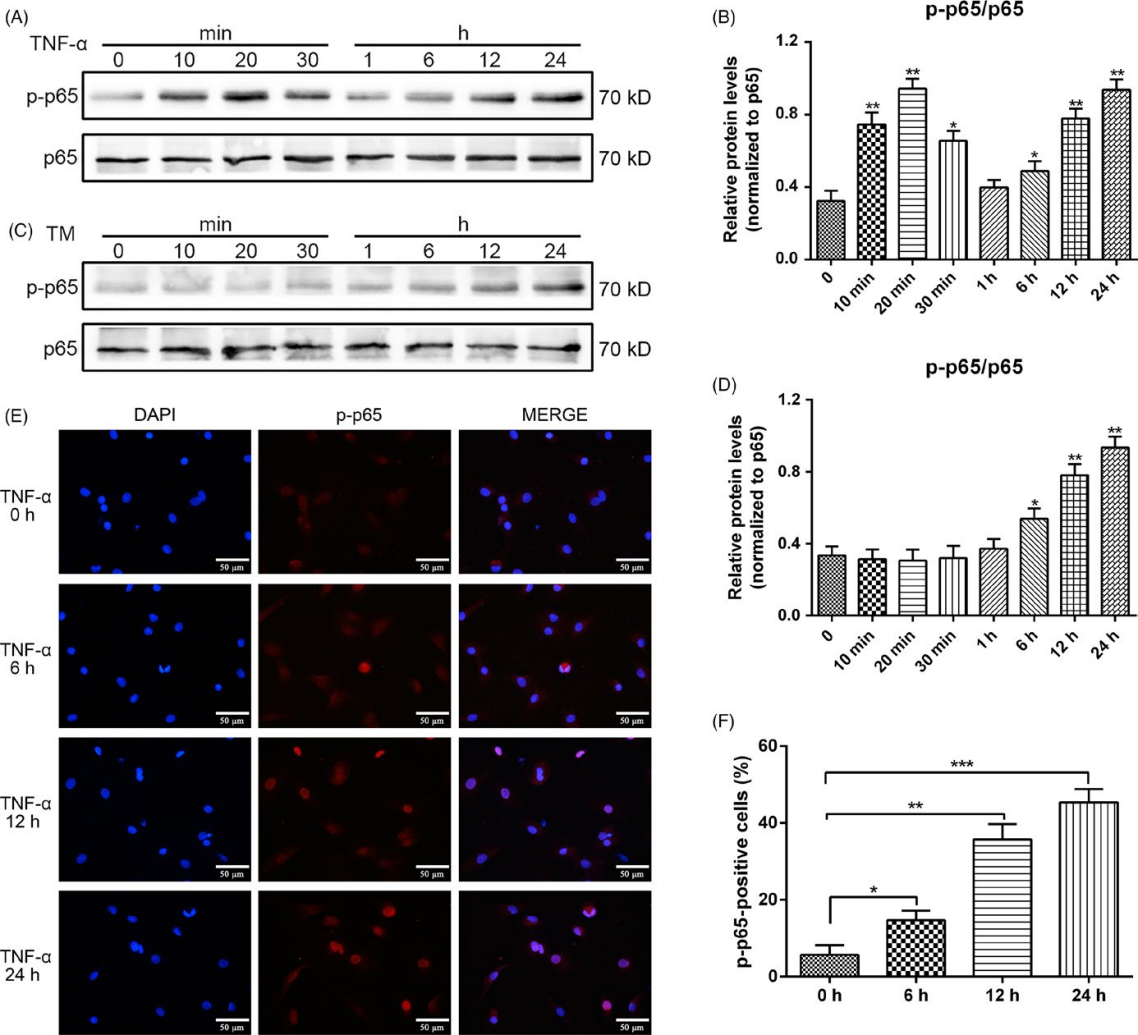
Kinetics of nuclear factor‐kappa B activation by tumour necrosis factor alpha (TNF‐α) and tunicamycin (TM). Nucleus pulposus cells (NPCs) were treated with either 10 ng/mL TNF‐α or 100 ng/mL TM at various time points. A,B, p65 protein phosphorylation with TNF‐α treatment was analysed through Western blot, and proteins were extracted to detect the expression of *p*‐p65. C,D, p65 protein phosphorylation with TM treatment was examined through Western blot. The protein expression levels of *p*‐p65 were normalized to that of p65. E, The nuclear translocation of *p*‐p65 under the TNF‐α stimulus was subjected to immunofluorescence studies (original magnification, ×400). F, *p*‐p65‐positive cells in the nucleus were quantified. The results were presented as mean ± SD (*n* = 3). *P < 0.05; **P < 0.01; ***P < 0.001

The branches of UPR have a crosstalk with the NF‐κB pathway.^13^ In this study, we aimed to validate whether the activation of UPR triggered the NF‐κB signalling in NPCs. We treated the NPCs with tunicamycin (TM), a widely used and recognized ER stressor that inhibits protein *N*‐glycosylation.^26^, ^27^ The activation of NF‐κB by TNF‐α is rapid, often occurring within minutes following stimulation. By contrast, ER stress and UPR are slow, cumulative process that increases as additional proteins accumulate in the ER over time. In our study, NF‐κB was rapidly induced by TNF‐α. An increase in the protein expression of *p*‐p65 was detected as early as 10 minutes after the treatment. The maximal activation occurred 20 minutes after stimulation; after 30 minutes, the *p*‐p65 expression was markedly reduced, and the expression increased again at 12‐24 hours (Figure 4A,B). By contrast, TM‐induced NF‐κB activation initially became detectable 12 hours after the treatment, increased further with time and reached high levels at 24 hours (Figure 4C,D). The transfer of *p*‐p65 from the cytoplasm to the nucleus is considered as a vital signal of the activation of NF‐κB signalling. Thus, we utilized immunofluorescence staining to investigate the translocation of *p*‐p65 into the nucleus. The results showed that additional *p*‐p65 translocated into the nucleus at 12 and 24 hours compared with that at 0 and 6 hours under TNF‐α stimulus (Figure 4E,F). We hypothesized that the slow and steady increase in the kinetics of TNF‐α‐induced NF‐κB activation after 12 hours was activated by the initiation of UPR.

### 3.5 TNF‐α‐mediated NF‐κB induction in the later phase was inhibited by XBP1 siRNA

**Figure 5:**
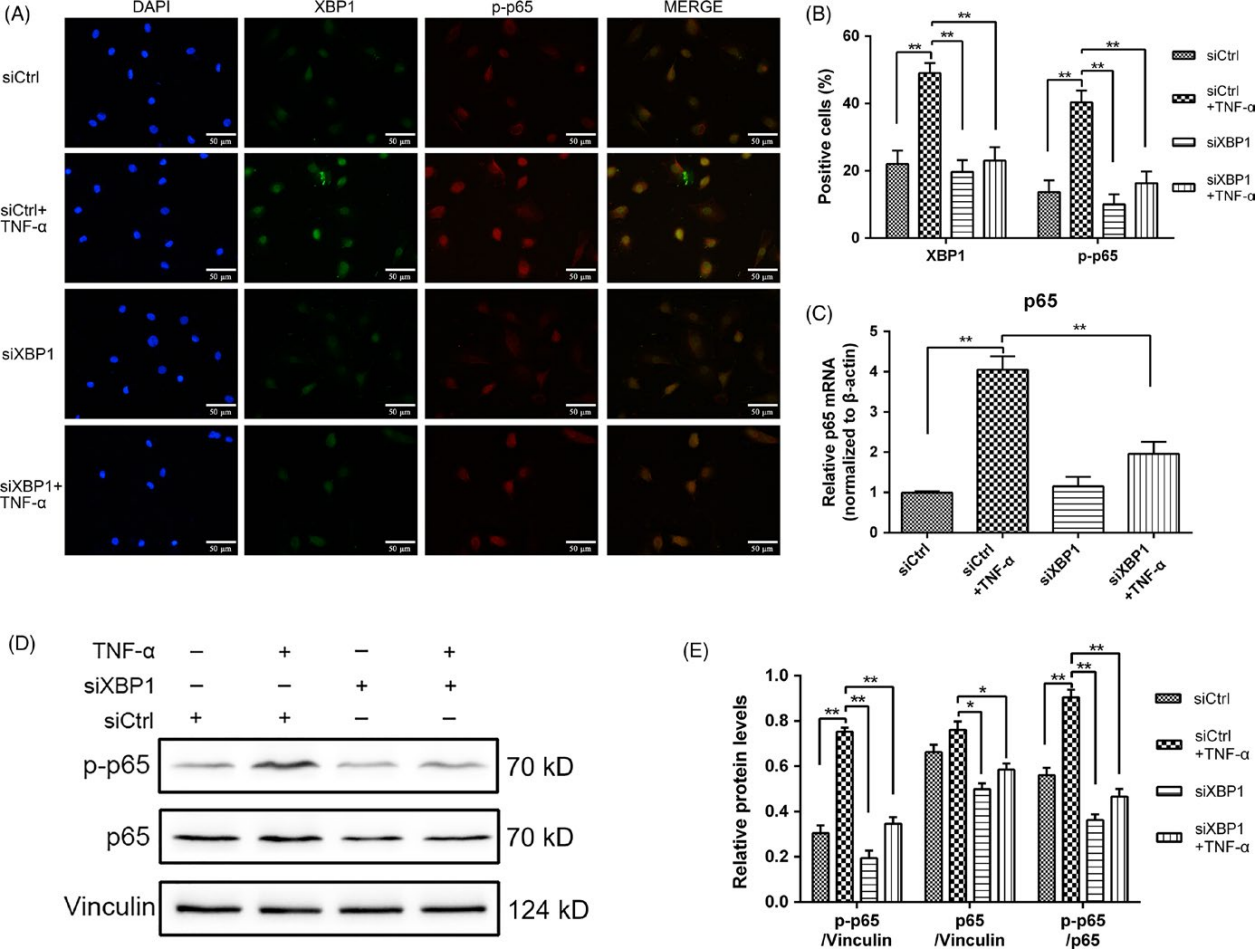
X‐box binding protein 1 (XBP1) regulates nuclear factor‐kappa B signalling by regulating p65 transcription and *p*‐p65 expression. The cells were exposed to 10 ng/mL tumour necrosis factor alpha with or without pretreatment with siCtrl and siXBP1 for 24 h. A, In immunofluorescence double staining, the cells were stained initially with XBP1 antibody, Alexa Fluor 488 (green), *p*‐p65 antibody and Alexa Fluor 647 (red), and the nucleus was counterstained with DAPI (blue) and examined through fluorescence microscopy (original magnification, ×400). B, XBP1‐ and *p*‐p65‐positive cells were quantified. C, Total RNA was extracted, and the mRNA expression of p65 was measured through qRT‐PCR. D,E, Proteins were extracted to detect the expression of *p*‐p65 and p65 through Western blot analysis, and the protein expression levels of *p*‐p65 were normalized to that of p65. The results were presented as mean ± SD (*n* = 3). *P < 0.05; **P < 0.01

To determine the underlying interaction between XBP1 and NF‐κB signalling and verify our speculations of NF‐κB activity regulation by XBP1, we silenced XBP1 with siRNA in rat NPCs. We performed immunofluorescence staining assay and found in the immunofluorescence images that *p*‐p65 was localized in the cytoplasm under siCtrl treatment. With TNF‐α treatment, *p*‐p65 translocated to the nucleus. XBP1 siRNA not only silenced XBP1 signalling, but also inhibited the nuclear translocation of *p*‐p65 (Figure 5A,B). The mRNA level of p65 was lower in the XBP1 siRNA‐treated cells than in the siCtrl‐treated cells under TNF‐α stimulation (Figure 5C). Furthermore, Western blot analysis showed that the knockdown of XBP1 with siRNA decreased the p65 and *p*‐p65 protein level and also decreased the value of *p*‐p65/p65 considerably, thereby suggesting that XBP1 regulated NF‐κB signalling in NPCs (Figure 5D,E).

### 3.6 NF‐κB signalling regulated the TNF‐α‐induced proliferation of NPCs

**Figure 6:**
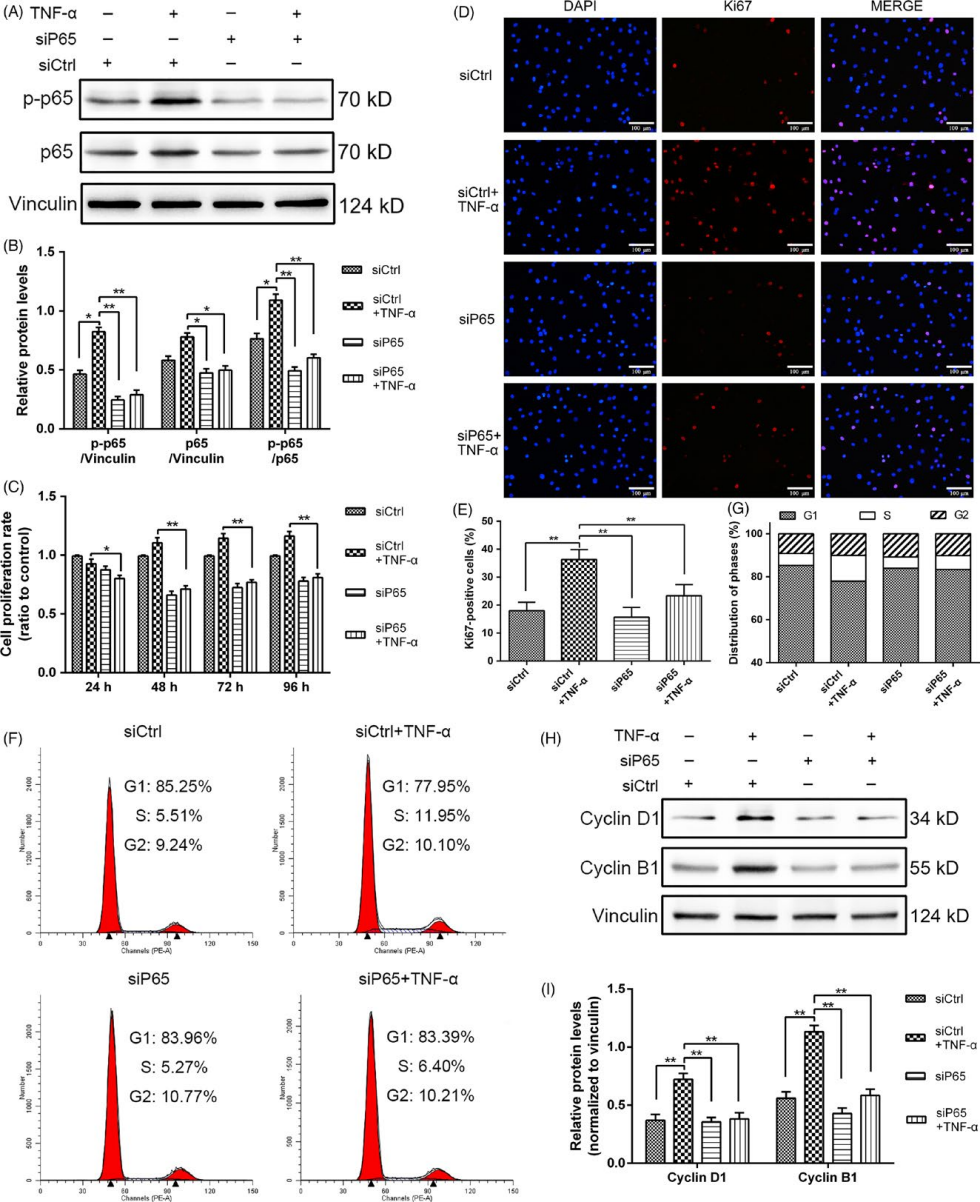
Role of nuclear factor‐kappa B (NF‐κB) signalling in rat NPC proliferation induced by tumour necrosis factor alpha (TNF‐α). The cells were exposed to 10 ng/mL TNF‐α with or without p65 siRNA (siP65) pretreatment for 24 h. A,B, NF‐κB‐p65 interference was verified through Western blot analysis. Proteins were extracted to detect the expression of *p*‐p65 and p65. The expression levels of *p*‐p65 were normalized to that of p65. C, Cell proliferation was evaluated through CCK‐8 analysis at different time points. D,E, Immunofluorescence staining of Ki67 (original magnification, ×200) and quantification of Ki67‐positive cells. F,G, The proportions of cells in each phase were examined through flow cytometry. H,I, Cyclin D1 and cyclin B1 expression levels in NPCs subjected to different treatments were measured through Western blot analysis and normalized to that of vinculin. The results were presented as mean ± SD (*n* = 3). *P < 0.05; **P < 0.01

To elucidate the connection of NF‐κB signalling and TNF‐α‐induced NPC proliferation, we knocked down the NF‐κB pathway through RNA interference. The silencing by p65 siRNA was verified by the decreased expression levels of p65 and *p*‐p65 proteins compared with that of siCtrl (Figure 6A,B). After the treatment with TNF‐α (10 ng/mL) or p65 siRNA was administered, the proliferation rate of NPCs was measured through CCK‐8 assay, and p65 silencing decreased the TNF‐α‐induced NPC proliferation at different time points (Figure 6C). We also detected the proliferating antigen Ki67 through immunofluorescence staining. The decreased nuclear expression of Ki67 was observed in the cells treated with p65 siRNA (Figure 6D,E), indicating a positive effect of NF‐κB signalling on NPC proliferation. Similarly, cell cycle analysis demonstrated that the knockdown of p65 increased the proportion of NPCs in the G1 phase and decreased the proportion of the cells that entered the S phase (Figure 6F,G). The protein expression levels of cyclin D1 and cyclin B1 were detected through Western blot analyses (Figure 6H,I). The results revealed that NPCs proliferated slowly under TNF‐α stimulation and expressed cyclin D1 and cyclin B1 to a lesser extent than that of normal cells after siRNA blocked p65 phosphorylation. Hence, NF‐κB signalling regulated the TNF‐α‐induced proliferation of NPCs.

## 4 DISCUSSION

In our observation, the acute exposure of TNF‐α increases the proliferation of rat NPCs and activates UPR signalling and NF‐κB signalling. In our previous studies, we demonstrated that ER stress facilitates the survival and proliferation of NPCs under TNF‐α stimulus by activating UPR.^23^ To the best of our knowledge, our work was the first to link UPR to NF‐κB signalling in the proliferation of NPCs. We also confirmed the proliferative effect of XBP1 on NPCs under TNF‐α stimulation. Hence, UPR‐activated NF‐κB signalling and its association with cell proliferation were revealed in our present research.

However, TNF‐α increases considerably in degenerative IVD. Inflammatory responses and cellular loss elicited by TNF‐α are considered as critical events in the progress of IDD and associated LBP,^28^ whereas in our model TNF‐α enhances NPC proliferation. The reasons may be as follows: first, the treatment of TNF‐α is a transient stimulation in our study, and some NPCs underwent apoptosis indeed within 24 hours. However, after 24 hours, the proliferation of surviving cells accelerated. Under acute TNF‐α exposure, NPCs with well stress capability initiating UPR, the UPR further maintains cellular homeostasis via activating downstream target genes and enhances cell proliferation through NF‐κB signalling, whereas the cells in poor stress statuses may apoptosis rapidly under TNF‐α stimulus. Second, IVD degeneration is a complicated and multifactorial process. The microenvironment of ischaemic and anoxia in IVD makes NPCs usually being stressful, and ER stress is easy to be induced in NPCs. ER stress is a double blade in both prosurvival and proapoptotic signalling. ER stress acts to restore normal ER homeostasis and is therefore cytoprotective. However, when the intensity of strong and prolonged TNF‐α stimulation exceeds the protein folded capacity of NPC, the cells can initiate apoptosis through ER stress or other pro‐apoptosis pathways.^29^ Third, the anabolism of proteoglycans (PGs) and collagens is accompanied by the proliferation of NPCs in our research. But, under persistent TNF‐α stimulation, the expression of MMPs and ADAMTSs is significantly upregulated in IVD tissue and cells.^30^ These increased matrix degradation enzymes can cleave collagens and PGs, especially COL2 and ACAN. Consequently, the imbalance between the catabolism and anabolism may lead to the ECM degradation and IDD.^31^ Based on all above these, our in vitro experiments may have different results with other reports.

Various genetic or environmental conditions that perturb the ER lead to misfolded protein accumulation in a condition known as ER stress. In response, cells adapt to such stress via subsequent signalling pathways, including UPR.^32^ UPR copes with disorders and restores ER homeostasis. When ER stress happens, BiP dissociates from three ER membrane sensors, namely IRE1, PERK and ATF6.^33^ IRE1 induces the splicing of XBP1, and PERK phosphorylates eIF2α. ATF6 becomes cleaved at the Golgi apparatus and induces ER‐associated degradation. These phenomena consequently inhibit the synthesis of unfolded proteins and shift cells to a prosurvival mode.^34^

XBP1, a key mediator of UPR, is activated by IRE1‐mediated splicing and implicated in cell proliferation. When ER stress occurs, the consequent IRE1 activation splices the mRNA of unspliced XBP1 (XBP1u). This splicing event results in the formation of an active XBP1s, which refolds the misfolded proteins. A functional study has demonstrated that XBP1 promotes cell growth and XBP1‐mediated cancer cell proliferation by upregulating matrix metalloproteinase‐9.^35^ Some studies have shown that XBP1s overexpression prevents cell cycle arrest in breast cancer cell lines and that XBP1 may participate in cell cycle regulation.^36^ Indeed, XBP1 is an important regulator of cell cycle and proliferation. In our study, to evaluate the hypothesis that TNF‐α induces the NPC proliferation in cooperation with the IRE1/XBP1 pathway, we blocked XBP1 signalling by using siRNA. The results confirmed that the knockdown of the XBP1 pathway weakened the proliferation of TNF‐α‐treated NPCs. As such, we considered that XBP1‐regulated NPC proliferation functioned in TNF‐α‐enhanced NP cell proliferation in vitro.

Extensive crosstalks occur between the UPR pathways and NF‐κB signalling via various mechanisms.^33^ All three branches of UPR may show potential for the induction of NF‐κB activation.^19^ Although PERK and ATF6 activate NF‐κB,^37^, ^38^ IRE1/XBP1 interference can evidently reduce p65 phosphorylation, indicating that the IRE1/XBP1 branch is the main pathway in UPR‐associated NF‐κB activation. Once UPR is activated, the phosphorylated IRE1 binds to TRAF2, leading to the phosphorylation and degradation of IκB and the nuclear translocation of NF‐κB.^33^, ^39^

It is well known that inflammation is closely related to cell viability and cell proliferation.^40^ NF‐κB plays an important role in cell proliferation as the key regulator of inflammation. Besides that, it has been proved that there are extensive crosstalks between inflammation and UPR.^41^ Inflammation can activate UPR via the oxidative stress of excessive protein folding. And UPR can initiate inflammation by regulating NF‐κB through IRE1 or PERK pathway. In addition, we proved that the UPR promoted NPCs proliferation through NF‐κB signalling. Hence, NF‐κB acts as a crucial link between inflammation and UPR, and they might interact and synergize to regulate cell proliferation.

TNF‐α can activate NF‐κB in many cell types. In this research, we detected the critical biomarker *p*‐p65 of TNF‐α‐treated NPCs, and the results revealed that TNF‐α could activate the NF‐κB signalling of NPCs via p65 phosphorylation at different time points. Previous studies confirmed that TNF‐α‐induced NF‐κB activation represents a simple, early, fast and effective response.^42^ In our research, TNF‐α induced early‐ and later‐phase NF‐κB activation, whereas TM could trigger late‐phase NF‐κB activation. Therefore, in the late phase, UPR activation might act as a trigger of NF‐κB. To examine our hypothesis, we blocked the XBP1 pathway with siRNA in NPCs and assessed the expression of the NF‐κB signalling component p65. Our results revealed that XBP1 siRNA suppressed TNF‐α‐induced NF‐κB activation in the late phase through the inhibition of p65 phosphorylation and nuclear translocation.

One research reported that NF‐κB‐p65 activation promoted vascular smooth muscle cell proliferation under inflammation through the microRNA‐17/RB pathway.^43^ Zhang et al^6^ found that the PAK5‐mediated phosphorylation and nuclear translocation of NF‐κB‐p65 promote the proliferation of breast cancer cells in vitro and in vivo. In our study, to demonstrate the hypothesis that TNF‐α‐induced NPC proliferation associated with the NF‐κB pathway, we knocked down NF‐κB‐p65 with p65 siRNA and found that cells blocking the p65 pathway proliferated more slowly than the control cells did under TNF‐α stimulus. All of these studies confirmed that NF‐κB‐p65 signalling was closely related to cell proliferation.

**Figure 7:**
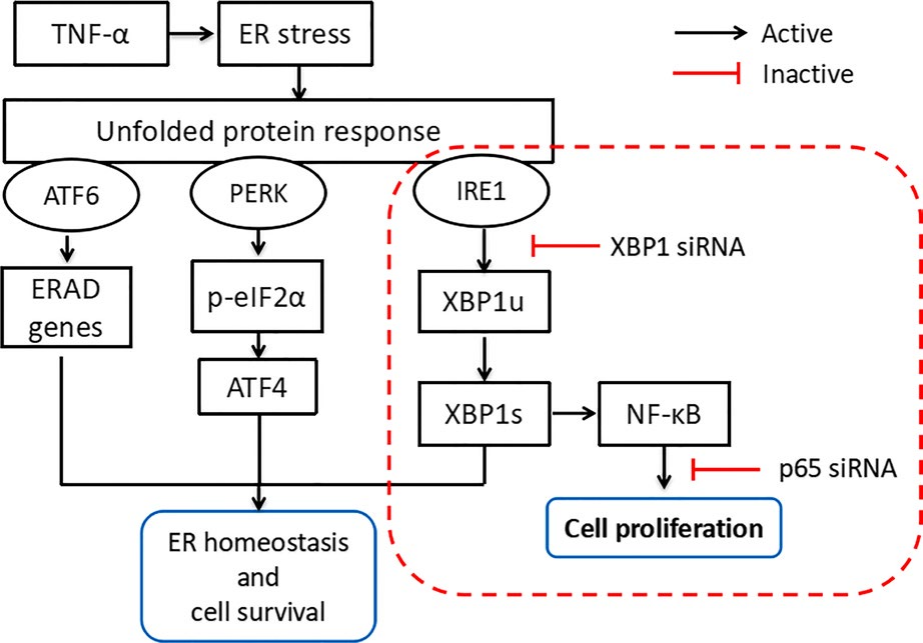
Schematic diagram proposed by the present research. Tumour necrosis factor alpha‐α‐mediated IRE1/XBP1 pathway induces NPC proliferation via NF‐κB signalling. ATF4, activating transcription factor 4; ATF6, activating transcription factor 6; eIF2α, eukaryotic translation initiation factor 2α; ER, endoplasmic reticulum; ERAD, ER‐associated degradation; IRE1, inositol‐requiring enzyme 1; PERK, protein kinase RNA‐like ER kinase; XBP1, X‐box binding protein 1

Overall, TNF‐α regulated the proliferation of NPCs through the IRE1/XBP1 pathway. Considering the relationship between IRE1/XBP1 and NF‐κB pathways, we concluded that NF‐κB signalling was essential for XBP1‐mediated NPC proliferation under TNF‐α stimulus (Figure 7).

## 5 CONCLUSIONS AND PERSPECTIVES

Our experiments confirmed that XBP1 signalling was effective in activating NF‐κB signalling by upregulating *p*‐p65 expression and nuclear translocation. A regulatory connection between XBP1 and NF‐κB survival signalling was determined. The proliferation of NPCs under TNF‐α stimulation was regulated via the NF‐κB‐dependent IRE1/XBP1 pathway. This study suggested that the XBP1/NF‐κB signalling could be a novel biological therapeutic target for the treatment of the cellular loss of NPCs under inflammation during IVD degeneration.

## CONFLICT OF INTEREST

The authors have declared that no competing interests exist.

## AUTHOR CONTRIBUTIONS

Lu Chen, Lei Liu and Xiao‐Tao Wu conceived and designed the experiments; Lu Chen, Zhi‐Yang Xie, Lei Liu, Feng Wang, Pan Fan, Arjun Sinkemani and Cong Zhang performed the experiments; Lu Chen, Lei Zhu and Lei Liu analysed the data; Lu Chen wrote the paper; and Lu Chen, Lei Zhu, Zhi‐Yang Xie, Xin Hong and Xiao‐Tao Wu reviewed and revised the manuscript. All authors read and approved the manuscript.
