# Supplementary figures and images for: Nuclear factor‐kappa B‐dependent X‐box binding protein 1 signalling promotes the proliferation of nucleus pulposus cells under tumour necrosis factor alpha stimulation
Source: Cell Prolif. 2018 Nov 14;52(2):e12542. doi: 10.1111/cpr.12542 (PMC6496019; doi:10.1111/cpr.12542)

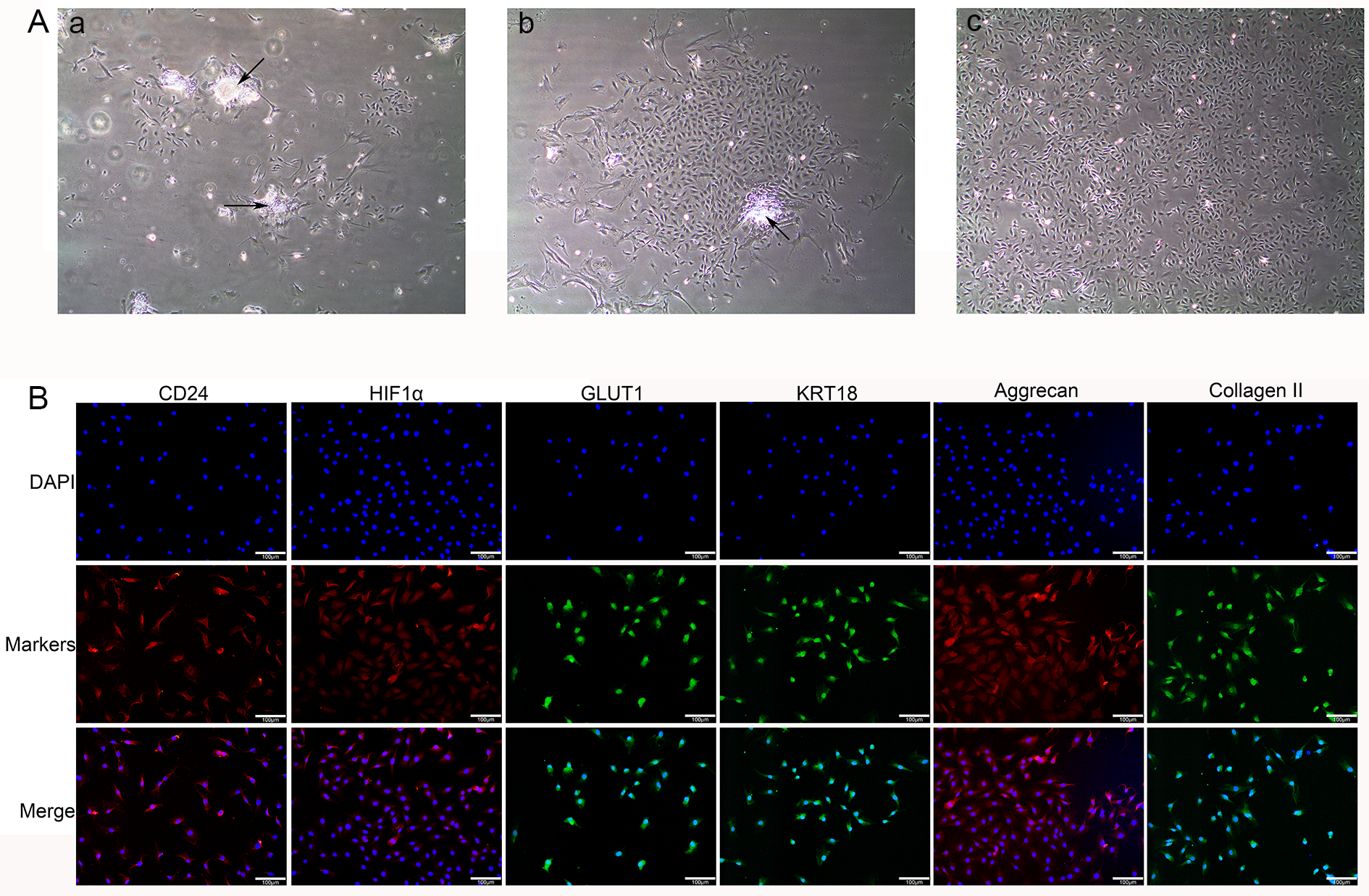

Supplement: Supplementary file 1 [file CPR-52-e12542-s001.tif]
