# Supplementary material for: Nuclear factor‐kappa B‐dependent X‐box binding protein 1 signalling promotes the proliferation of nucleus pulposus cells under tumour necrosis factor alpha stimulation
Source: Cell Prolif. 2018 Nov 14;52(2):e12542. doi: 10.1111/cpr.12542 (PMC6496019; doi:10.1111/cpr.12542)
Supplement: Supplementary file 2 [file CPR-52-e12542-s002.docx]

**Supplemental Figure.** NPCs identification. (A) Morphology of NPCs in monolayer cultures (original magnification, x40). (a) Cell masses of primary NPCs. The cytoplasm-vacuolated notochordal cells (arrow) were seen in the primary NPCs. (b) NPCs migrated out of the partially digested NP tissues (arrow). (c) NPCs at passage 3. The cytoplasm-vacuolated notochordal cells gradually disappeared. (B) Detection of the NPC phenotypic markers through immunofluorescence staining (original magnification, x200).
